# Cytotoxic and inflammatory potential of size-fractionated particulate matter collected repeatedly within a small urban area

**DOI:** 10.1186/s12989-015-0099-z

**Published:** 2015-07-16

**Authors:** Errol M. Thomson, Dalibor Breznan, Subramanian Karthikeyan, Christine MacKinnon-Roy, Jean-Pierre Charland, Ewa Dabek-Zlotorzynska, Valbona Celo, Prem Kumarathasan, Jeffrey R. Brook, Renaud Vincent

**Affiliations:** Inhalation Toxicology Laboratory, Hazard Identification Division, Environmental Health Science and Research Bureau, Health Canada, 0802B Tunney’s Pasture, Ottawa, ON K1A 0K9 Canada; Analysis and Air Quality Section, Air Quality Research Division, Atmospheric Science and Technology Directorate, Environment Canada, Ottawa, ON K1A 0H3 Canada; Mechanistic Studies Division, Environmental Health Science and Research Bureau, Health Canada, Ottawa, K1A 0K9 Canada; Air Quality Processes Research Section, Air Quality Research Division, Atmospheric Science and Technology Directorate, Environment Canada, Toronto, ON M3H 5T4 Canada

**Keywords:** Air pollution, Particulate matter, Sources, Industry, Traffic, Toxicity, Inflammation, Metals, Polycyclic aromatic hydrocarbons, Endotoxin

## Abstract

**Background:**

Exposure to coarse, fine, and ultrafine particles is associated with adverse population health impacts. We investigated whether size-fractionated particles collected repeatedly in the vicinity of industrial (steel mills and associated coking operations, wastewater treatment), high traffic, and residential areas display systematic differences in biological potency.

**Methods:**

Particulate matter (PM_<0.1_, PM_0.1–0.5_, PM_0.5–2.5_, PM_2.5–10_, PM_>10_) samples collected at sites within Windsor, Ontario, were screened for biological potency in human A549 lung epithelial and murine J774A.1 macrophage-like cells using cytotoxicity bioassays (cellular ATP, resazurin reduction, lactate dehydrogenase (LDH) release), cytokine production, and transcript profiles. Potency was determined from the slope of each dose-effect relationship.

**Results:**

Cytotoxic potency varied across size fractions and within a fraction across sites and sampling periods, suggesting that particle composition, in addition to size and mass, affected particle toxicity. While ATP and LDH profiles showed some similarity, resazurin reduction (a measure of metabolic activity) exhibited a unique pattern of response, indicating that the cytotoxicity assays were sensitive to distinct particle characteristics. Chemical speciation varied in relation to prevailing winds, consistent with enrichment of source emissions (e.g. higher metal and polycyclic aromatic hydrocarbon content downwind of the industrial site). Notwithstanding this variability, site-dependent differences in particle toxicity were evident, including greater potency of coarse fractions at the industrial site and of ultrafine particles at the traffic site (*Site* × *Size* interactions, *p* < 0.05). Regression of potency against particle constituents revealed correlations between resazurin reduction, induction of metal-responsive genes, and metal content, which were particularly strong for the coarse fraction, and between cytokine release and endotoxin, suggesting that these factors were important drivers of biological effects that explain, at least in part, the contrasting potencies of particles compared on an equivalent mass basis.

**Conclusions:**

The data show that 1) particle potency and composition can exhibit significant temporal variation in relation to source contributions; 2) sources may differentially impact the potency of specific size fractions; and 3) particle constituents, notably metals and endotoxin, may elicit distinct biological responses. Together, the data are consistent with the notion that sources and composition, in addition to size and mass concentration, are relevant to particle toxicity.

**Electronic supplementary material:**

The online version of this article (doi:10.1186/s12989-015-0099-z) contains supplementary material, which is available to authorized users.

## Background

Airborne particulate matter (PM) levels are associated with pulmonary and cardiovascular morbidity and mortality [1–3]. Although current air quality guidelines are based on mass concentration of particles of a given aerodynamic diameter (e.g. PM_2.5_), it is clear that size is not the sole determinant of hazard, and indeed variation in the magnitude of effects is observed in population studies according to location and season [4, 5]. Health effects of ambient particulate matter may be due to complex combinations of chemical constituents (e.g. metals, organic species) and physical properties (e.g. size, surface area) that vary spatially and temporally, act through diverse biological pathways, and interact with host-specific traits (e.g. existing disease state, gene polymorphisms) to provoke adverse effects in target cells and tissues. A greater understanding of how sources and specific constituents differentially impact particle toxicity is needed for targeted regulation of those emissions most relevant to adverse health effects [6].

Local industrial point sources, commercial and residential area sources, transportation sources, and long-range transport collectively contribute to the composition of airborne particles at any given site and time, and may differentially affect the toxicological properties of the mixture. Analysis of intra-urban variability in exposure levels has shown that the relative risk of adverse health effects may exceed the relative risk observed through inter-city comparisons [7, 8]. Proximity to emission sources may also impact particle potency as a result of enrichment of source contributions. Particles collected in urban or rural settings or in the vicinity of traffic or industrial sources display a range of toxic and inflammatory potencies in relation to size and composition that point to the importance of sources in determining particle toxicity [9–12]. Repeated sampling of PM in New York City over a number of 48–72 h periods produced samples that exhibited a range of oxidative potencies [13], suggesting that monitoring the temporal variability of particle toxicity could provide valuable data on the relative importance of contributions from specific sources.

Windsor, Ontario and Detroit, Michigan are neighbouring cities that together constitute an important centre for industrial activity and international trade between Canada and the United States. Because of the proximity of the two urban centres and the presence of heavy industry and high levels of traffic, for decades the issue of cross-boundary pollution and adverse health effects has received attention [14–17]. Air quality in Windsor is impacted both by domestic and international sources, including industrial point sources in both Windsor and Detroit, and heavy traffic associated with the international Ambassador Bridge. Recent studies conducted in the area have examined associations of pollutants with respiratory and cardiovascular health outcomes, including pulmonary function [18] and exacerbation of asthma [19] in children, acute incidence of asthma [17, 20], and cardiovascular parameters in the elderly [21]. Intra-urban variations in air pollutant levels in Detroit and Windsor were found to correlate with asthma rates in the area defined by 3-digit postal code [17], suggesting that variability in exposures within the urban area contributes to spatial variability in adverse health outcomes.

Exposure to coarse, fine, and ultrafine particles is associated with adverse health effects in the human population, but effects may differ according to size fraction, composition, and target tissues. A better understanding of the relative importance of specific physicochemical characteristics of particles in provoking adverse health effects in humans will require convergence of results from controlled toxicological investigation (both *in vitro* and *in vivo*) and epidemiological studies. Cell culture assays provide a means for rapid screening of particles for biological potency, and for identification of toxicity determinants. While many *in vitro* studies have examined the relationship of particle physicochemical characteristics and toxicity, there is little consensus on the relation between composition (and hence source) of environmental samples and particle potency. Moreover, comparisons of particles collected in the vicinity of different sources are generally based on a single sample per site, which does not allow assessment of the temporal variability in potency or composition at a given location resulting from differential enrichment of source contributions. In the present study, we compared the relative biological potency of size-fractionated particles collected in the vicinity of residential, industrial, and high traffic sites within a small urban area in Windsor, Ontario, Canada. Particles were sampled repeatedly at each site to assess the temporal variability in particle toxicity and strengthen the association of toxicity with source. Biological effects were assessed across a range of doses in two well-characterised cell lines (epithelial, macrophage-like) employed previously for assessment of particle toxicity (e.g. [22, 23]) using a panel of assays to capture possible complexity in the response. The principal objective was to examine whether size-fractionated particles collected at sites located close to sources (industrial, transportation, urban background) display systematic differences in toxicity. We show that particles compared on an equivalent mass basis exhibit considerable variability in potency at a given site, that sites display systematic differences in particle toxicity, and that the potency contrasts can be explained, at least in part, by levels of specific particle constituents.

## Results

### Particle collection

**Fig. 1:**
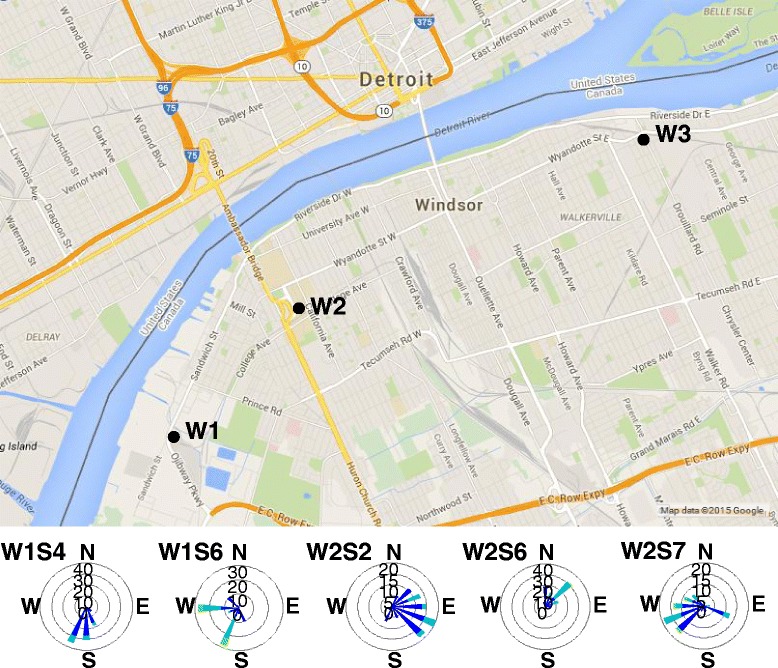
Location of particle sampling sites (W1, industrial; W2, traffic; W3; urban background) in Windsor, Ontario, and wind roses for a subset of sampling periods. Wind roses are labelled according to sampling period (e.g. W1S1 indicates sample 1 collected at site W1). Vectors represent the proportion of a collection period that the wind was coming from a given direction. Wind speed is displayed in m/s (*dark blue*, 1–3 m/s; *light blue*, 4–6 m/s; *green*, 7–10 m/s). The complete set of wind roses is presented in Additional file 1: Figure S1

Size-fractionated particles were collected at three sites: a site close to industrial sources including a wastewater treatment plant, major steel mills, and associated coking operations (W1); a second site (W2) close to intensive traffic crossing the Ambassador Bridge including heavy-duty diesel engines; and a third site (W3) representative of a general urban area selected for comparison to the more source-impacted sites (Fig. 1). Wind roses employed as a means of relating particles to potential sources confirmed that the sampling campaign captured periods during which the prevailing wind came from a variety of directions (Fig. 1; complete set displayed in Additional file 1: Figure S1). PM_2.5_ levels measured at the two local National Air Pollutant Surveillance (NAPS) monitoring stations were used to characterise ambient conditions during periods of collection. Measurements at these two stations were highly correlated (*r* = 0.88; *r* = 0.99 after removal of one outlier), and thus their averages, determined separately for each period of collection, were calculated. Because vandalism at the urban background site resulted in only one sample having a known collection period, we only compared NAPS data during the collection periods for the industrial site and the traffic site here. However, particle samples from the urban background site were available for toxicity assays. On average, PM_2.5_ levels determined by the NAPS station data did not differ between the collection periods for the industrial and traffic sites, but varied considerably during collection periods for the individual ChemVol samples (industrial site average 11.5 ± 9.3 μg/m^3^, range 5.9–31.8 μg/m^3^; traffic site average 11.2 ± 5.4 μg/m^3^, range 6.1–19.9 μg/m^3^).

### Cytotoxicity screening

**Fig. 2:**
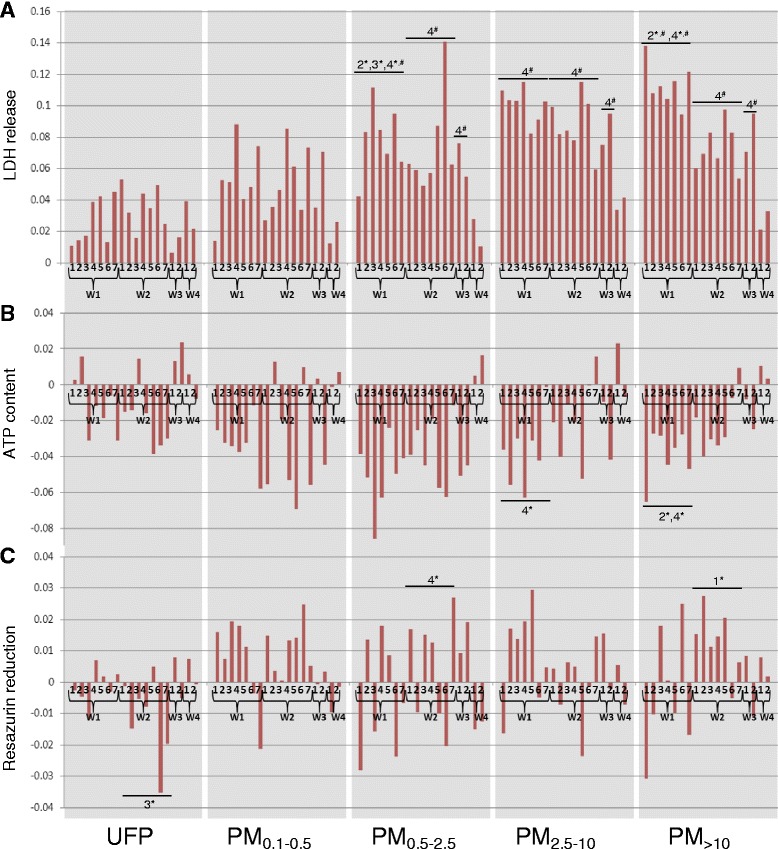
Cytotoxic potency of size-fractionated particles from the industrial (W1), traffic (W2), and urban background (W3) sites in macrophage-like J774 cells. **a** Membrane integrity (lactate dehydrogenase release). **b** Cellular energy state (ATP content). **c** Cellular redox status (resazurin reduction). Potency estimates were calculated using the results of three independent experiments, and represent the slope of the dose-effect relationship. W4 represents exposures to extracts of field blanks. Statistical analyses were performed on both the cytotoxicity data and the potency data, as described in Materials and Methods. For simplicity, only effects from significant *Site × Size* interactions are presented. Numbers above bars indicate that samples are statistically different from that group according to post-hoc pairwise multiple comparison (e.g. “2, 4” above group W1 signifies that W1 is different from groups W2 and W4, Holm-Sidak method, *p* < 0.05), with asterisks denoting a significant effect according to analysis of the raw cytotoxicity data and pound signs denoting a significant effect according to analysis of potency data. *Site* × *Size* interactions for potency data: LDH, *p* = 0.01; ATP, *p* = 0.374; resazurin reduction, *p* = 0.168; for cytotoxicity data: LDH, *p* < 0.001; ATP, *p* = 0.026; resazurin reduction, *p* = 0.013

**Fig. 3:**
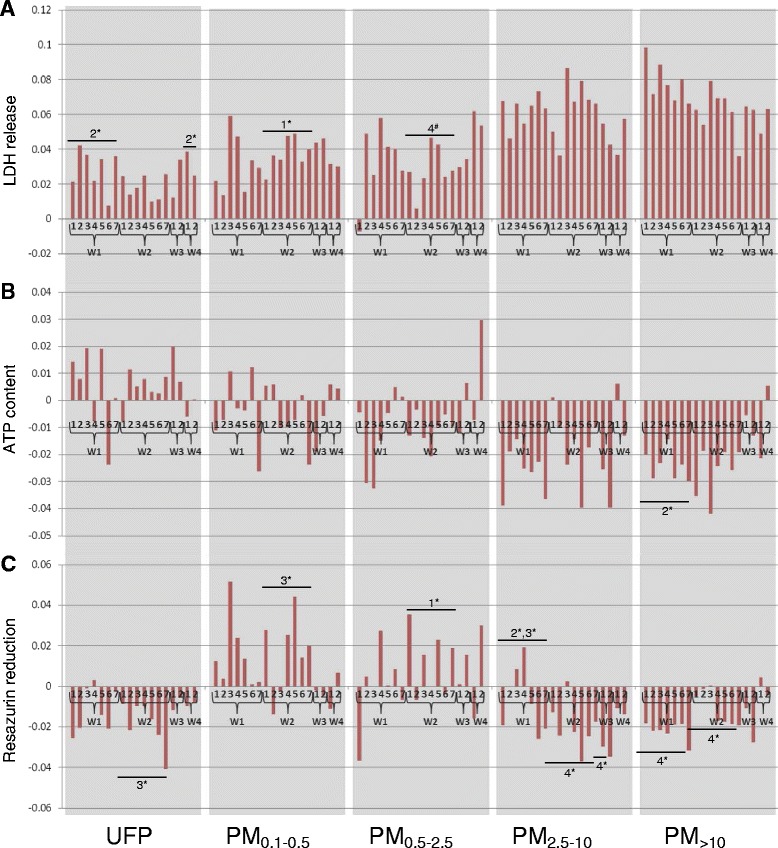
Cytotoxic potency of size-fractionated particles from the industrial (W1), traffic (W2), and urban background (W3) sites in lung epithelial A549 cells. **a** Membrane integrity (lactate dehydrogenase release). **b** Cellular energy state (ATP content). **c** Cellular redox status (resazurin reduction). Potency estimates were calculated using the results of three independent experiments, and represent the slope of the dose-effect relationship. W4 represents exposures to extracts of field blank. Statistical analyses were performed on both the cytotoxicity data and the potency data, as described in Materials and Methods. For simplicity, only effects from significant *Site × Size* interactions are presented. Numbers above bars indicate that samples are statistically different from that group according to post-hoc pairwise multiple comparison (e.g. “2, 4” above group W1 signifies that W1 is different from groups W2 and W4, Holm-Sidak method, *p* < 0.05), with asterisks denoting a significant effect according to analysis of the raw cytotoxicity data and pound signs denoting a significant effect according to analysis of potency data. *Site* × *Size* interactions for potency data: LDH, *p* = 0.046; ATP, *p* = 0.138; resazurin reduction, *p* = 0.144; for cytotoxicity data: LDH, *p* = 0.003; ATP, *p* = 0.008; resazurin reduction, *p* < 0.001

Biological responses to particle exposure were assessed in macrophage-like J774 cells and in A549 lung epithelial cells. Particles exhibited concentration-dependent effects after 24 h exposure for all cytotoxicity assays, but the magnitude of effects varied considerably according to cell type, particle size, site, and sampling periods (Additional file 2: Figure S2 and Additional file 3: Figure S3 for J774 and A549 data respectively). The signal-to-noise ratio was considerably higher in J774 cells, as revealed by comparison of responses to particle samples with those to field blanks. To simplify the large dataset, potency estimates [24] were used to describe the exposure-response relationships in J774 and A549 cells (Figs. 2 and 3 respectively; complete statistical analyses presented in Additional file 4: Table S1). Briefly, data for each assay were expressed as fold-change relative to the mean of control (zero dose) samples, and a curve was fitted according to the equation Fold change = (1 + dose)^β^, with β (the slope of the dose-response curve) used as the measure of potency (β = 0 signifying no potency). Potency, as assessed by lactate dehydrogenase (LDH) release or by reduction of intracellular ATP, tended to increase with particle size in J774 (Fig. 2a and b) and A549 cells (Fig. 3a and b). While effects were correlated across cell lines (LDH, *r* = 0.6, *p* < 0.001; ATP, *r* = 0.36, *p* < 0.001), J774 cells showed greater sensitivity to fine PM_0.1–0.5_ and PM_0.5–2.5_ fractions (Fig. 2a and b). Effects of particles on metabolic activity (assessed by resazurin reduction) were distinct from those measured using LDH and ATP assays: in J774 cells there was no clear trend except possibly towards increased reactivity with increasing particle size (Fig. 2c), while in A549 cells, ultrafine, PM_2.5–10_, and PM_>10_ particles exhibited higher cytotoxic potency than fine particles (*Size* main effect, *p* < 0.001; Fig. 3c). While resazurin reduction was correlated between cell lines (*r* = 0.45, *p* < 0.001), it was only marginally correlated with ATP in A549 cells (*r* = 0.21, *p* = 0.04) and showed no significant relationship with LDH in either cell line.

Notwithstanding these general observations, statistical analyses revealed that the sampling site modified the effect of particle size in both cell lines (significant *Site* × *Size* interactions, described in Figs. 2 and 3 legends and in Additional file 4: Table S1). In J774 cells, PM_0.5-2.5_, PM_2.5–10_, and PM_>10_ fractions from the industrial site tended to be more potent than the respective size fractions from the traffic site according to the LDH and ATP assays (Fig. 2a and b). Ultrafine particles tended to be more potent at the traffic site according to the resazurin assay in either cell type (Figs. 2c and 3c).

### Inflammatory potential and RNA profiling

**Fig. 4:**
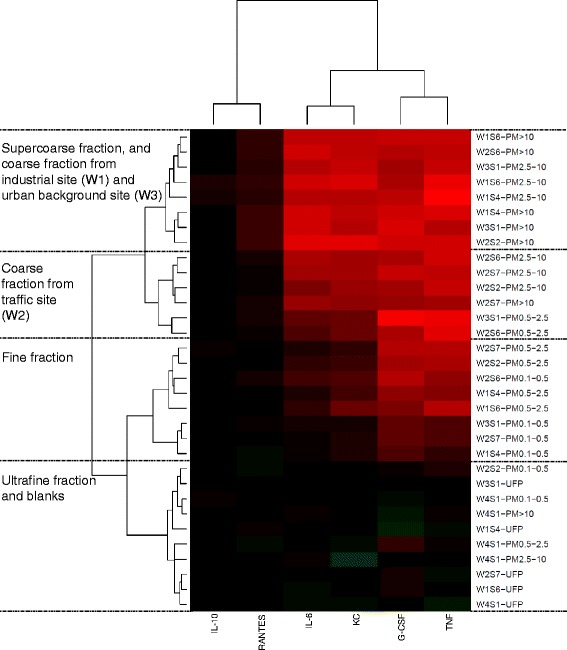
Hierarchical clustering of particles according to cytokine response in J774 cells. Potency estimates were calculated using the results of 3 independent experiments. The heat map displays particle potency estimates determined for all cytokines assayed that were above the limit of detection. Descriptions of the main contents of each cluster are included to the left of the plot. Red indicates increased expression, green indicates decreased expression. W1, industrial site; W2, traffic site; W3, urban background site; W4, field blanks

To further evaluate the relationship between sites, sizes, and biological effects, we examined cytokine production and RNA profiles of genes implicated in antioxidant and inflammatory responses in the macrophage-like J774 cell line using particles for which the wind roses suggested possible contrasts in source contributions (Fig. 1). Hierarchical clustering of particles according to cytokine potency produced groups that predominantly reflected particle size, with the coarse and super-coarse particles exhibiting the greatest potency, followed by the fine PM_0.5–2.5_ and PM_0.1–0.5_, while ultrafine particles did not elicit an inflammatory response (*Size* and *Site* main effects, *p* < 0.05; Fig. 4). PM_0.5–2.5_ from the traffic sample W2S6 and the urban background sample W3S1 clustered with larger size fractions the traffic site, while PM_0.1–0.5_ from W2S6 clustered with the remaining PM_0.5–2.5_ samples, indicating that these particles produced an inflammatory response somewhat dissimilar from other particles of the same size fraction and more in keeping with larger particles (i.e. were more inflammogenic). Statistical analysis of the dose-response data for individual cytokines identified *Site × Size* factor interactions for G-CSF, KC, TNF, and IL-6 (*p* < 0.001). Post-hoc pairwise comparisons identified statistically significant differences in responses to the PM_2.5–10_ fraction, indicating a greater response to samples from the industrial site compared to the other two sites for KC (W1 vs. W2, W3; *p* < 0.05) and TNF (W1 vs. W3; *p* = 0.048), and to the traffic site for IL-6 (W2 vs. W1, W3; *p* < 0.001), while PM_>10_ from both the industrial and traffic sites tended to be more inflammogenic than the urban background site according to TNF (W1, W2 vs. W3; *p* < 0.05). Pro-inflammatory cytokine/chemokine responses were highly correlated (*p* < 0.001 for all pairwise comparisons, Pearson correlations > 0.8 for most, data not shown). In contrast, the anti-inflammatory cytokine IL-10 did not exhibit any change in expression, while IL-1α and IL-13 were below the limit of detection (data not shown).

Similar to the cytokine response, mRNA transcript profiles tended to cluster according to particle size, with PM_2.5–10_ and PM_>10_ particles generally exhibiting the greatest potency while ultrafine particles clustered with the negative controls (Additional file 5: Figure S4). The PM_0.1–0.5_ fraction from traffic site sample W2S6 again exhibited greater potency than other samples of this size fraction, as did the PM_0.5–2.5_ fraction of traffic site sample W2S2, both clustering with particles of larger size fractions. Transcript levels of genes involved in inflammatory response (TNF, IL-6, endothelin-1, inducible nitric oxide synthase) were highly correlated (r > 0.8, *p* < 0.001), and showed some consistency with the cytokine data (e.g. r > 0.9 for correlation between TNF in supernatant and TNF mRNA). The metal and oxidative-stress responsive factors metallothionein (MT)-1 and −2 and the antioxidant heme oxygenase (HMOX1) were also significantly correlated with each other and with the inflammatory markers (correlation coefficients of 0.4–0.7). Of the four aryl hydrocarbon receptor-regulated genes analysed, only TCDD-inducible poly (ADP-ribose) polymerase (TiPARP) was expressed above background, and like the other genes its expression tended to vary with particle size.

### Integrated estimate of potency

**Fig. 5:**
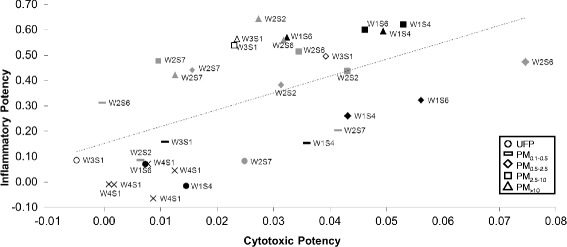
Overall potency of size-fractionated particles as a function of cytotoxic and inflammatory potencies in J774 cells. Potency estimates were calculated as described in Materials and Methods. Black fill, industrial site (W1); grey fill, traffic site (W2); white fill, urban background site (W3); X, field blank. Correlation coefficient, *r* = 0.59, *p* < 0.001

The cytotoxicity assays clearly yielded distinct response profiles, indicating sensitivity to different physicochemical characteristics of the particles. If all assays are considered equivalent, averaging responses across multiple assays may provide a more representative estimate of particle potency compared to results of any single assay. To generate an integrated estimate of particle potency, cytotoxic potency (calculated as the average of potencies for LDH release, resazurin reduction, and ATP content) was plotted relative to inflammatory potency (calculated as the average of all cytokine potencies) using data from J774 cells (Fig. 5). Cytotoxic and inflammatory potencies were positively correlated (*r* = 0.59, *p* < 0.001). Coarse and super-coarse particles were found in the upper part of the plot, indicating that they were generally the most inflammogenic but that they elicited a range of cytotoxic responses: particles from the industrial site (W1S4, W1S6) grouped in the upper right, indicating higher cytotoxicity, while samples from the traffic site (W2) exhibited similar inflammatory potential but lower cytotoxicity. PM_0.5–2.5_ fraction from the industrial site tended to be more cytotoxic but less inflammogenic than other particles of this size fraction, with the exception of sample W2S6 from the traffic site which exhibited elevated cytotoxic potency. The PM_0.1–0.5_ sample from the same site (W2S6) also displayed the highest inflammatory potential of its size fraction, but was less cytotoxic than most. Ultrafine particles generally clustered with the field blanks (W4) in the lower left, indicating generally low relative cytotoxicity and inflammatory potential.

### Particle composition and associations with biological responses

**Fig. 6:**
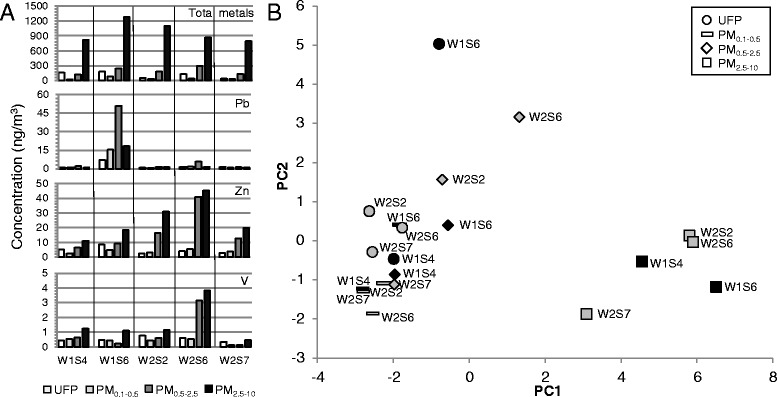
Metal composition of size-fractionated particulate matter. **a** Levels of total metals and selected metal concentrations (mass concentration per volume of air, ng/m^3^). A complete table of metal concentrations is presented in Additional file 6: Table S2. **b** Principal component analysis of particles according to metal composition. Black fill, industrial site (W1); grey fill, traffic site (W2)

As samples collected at a given site or at sites within close geographic proximity displayed differential cytotoxic and inflammatory potential, we investigated possible source-dependent differences in composition for the subset of samples selected on the basis of contrasting wind direction and/or potency. A table of all metals analysed is included in Additional file 6: Table S2. Larger fractions were composed primarily of aluminum and iron, whereas ultrafine particles had significant levels of titanium. There were several striking differences in particle composition across sites. W1S6 samples, collected over a period during which the prevailing wind was from the west, exhibited significantly higher levels of metals for most size fractions, including enrichment of lead (Fig. 6a) and copper across size fractions compared to other samples. In contrast, W1S4 particles, collected during a period of high particulate levels and wind predominantly from the south, tended to have lower overall metal concentrations, but with chromium notably increased in the ultrafine fraction while selenium was elevated in the fine fractions. The traffic sample W2S6 had relatively high levels of metals in the ultrafine and fine fractions compared to the other two traffic samples assayed, and enrichment of zinc, barium, and vanadium. Principal component analysis of the elemental composition resulted in groupings mainly according to particle size, with coarse particles separated from fine and ultrafine particles (Fig. 6b). The W1S6 ultrafine and W2S6 PM_0.5–2.5_ samples were separated from other ultrafine and fine particles by the second component driven by cadmium, arsenic, tellurium, titanium, and lead.

**Fig. 7:**
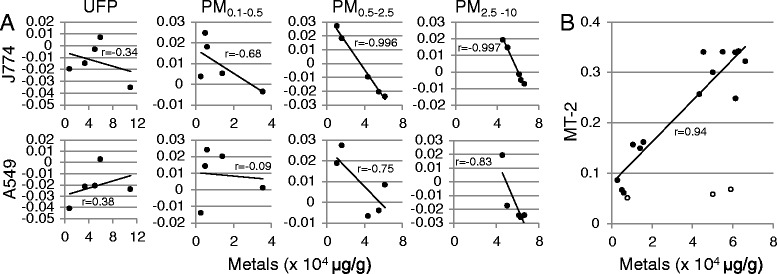
Association of biological effects with metal content in size-fractionated particles. **a** Correlation of total metals (μg/g extracted particle) with potency according to metabolic function (assessed by impacts on resazurin reduction) after 24 h exposure of A549 and J774 cells. **b** Association of metal content with potency according to metallothionein-2 (MT-2) mRNA in J774 cells after 4 h exposure. Potency estimates were calculated as described in Materials and Methods. Open circles, ultrafine particles; filled circles, all other size fractions (PM_0.1–0.5_, PM_0.5–2.5_, PM_2.5–10_). The trend line and correlation coefficient exclude ultrafine particles (*r* = 0.69 for all particles)

Regression of cytotoxic potency against total metal content revealed a significant negative correlation between total metals and resazurin reduction (J774, *r* = −0.60, *p* = 0.005; A549, −0.43, *p* = 0.059), but no significant association with LDH release or ATP levels (Table 1). To assess possible interactions between particle size and metals, results were stratified by particle size (Fig. 7a). In both cell lines, resazurin reduction following exposure to ultrafine particles appeared unrelated to total metal content. However, as particle size increased, resazurin reduction was increasingly correlated with metal content. Total metal content was also positively correlated with mRNA expression of the metal- and antioxidant response gene metallothionein-2 (*r* = 0.69, *p* = 0.002), with the correlation improving when ultrafine particles were assessed separately (Fig. 7b). Inflammatory cytokines tended to correlate with metal content (*r* = 0.44, *p* = 0.08), with IL-6 (*r* = 0.56, *p* = 0.02) showing the highest correlation. However, correlations between metals and cytokines appeared largely attributable to covariance of effects with increasing particle size, as the relationships did not hold when evaluated within each size fraction (Table 1).

**Table 1:** Pearson correlations for biological potency and particle constituents across size fractions in J774A.1 and A549 cell models

	Metals							Polycyclic aromatic hydrocarbons							Endotoxin						
	J774 cells				A549 cells			J774 cells				A549 cells			J774 cells				A549 cells		
Size	LDH	ATP	RES	CYT	LDH	ATP	RES	LDH	ATP	RES	CYT	LDH	ATP	RES	LDH	ATP	RES	CYT	LDH	ATP	RES
UFP	0.68	−0.24	−0.34	−0.72	−0.55	−0.21	0.38	−0.65	0.15	−0.17	0.79	−0.61	−0.54	−0.49	0.08	−0.23	−0.27	0.87	−0.03	0.07	−0.35
PM_0.1–0.5_	0.00	−0.18	−0.68	0.23	−0.32	0.26	−0.09	−0.68	0.46	−0.36	0.64	−0.89	0.52	−0.40	0.02	0.43	−0.27	0.67	0.14	−0.04	**−0.60** ^*^
PM_0.5–2.5_	0.56	−0.38	**−1.00** ^*^	0.15	−0.30	**0.86** ^**^	−0.75	−0.17	0.26	−0.68	0.01	−0.47	**0.93** ^*^	−0.59	0.19	0.39	**0.54** ^*^	0.27	**−0.44** ^**^	0.28	0.37
PM_2.5–10_	−0.13	0.02	**−1.00** ^*^	−0.42	−0.16	0.30	**−0.83** ^**^	−0.05	−0.46	−0.44	0.37	0.17	−0.31	−0.28	**0.54** ^*^	**0.49** ^**^	0.26	0.49	0.09	**0.55** ^*^	−0.15
ALL	0.23	−0.16	**−0.60** ^*^	**0.44** ^**^	−0.07	−0.15	**−0.43** ^**^	0.32	−0.15	−0.26	**0.63** ^*^	0.28	−0.24	−0.29	**0.70** ^*^	**0.21** ^**^	−0.06	**0.89** ^*^	0.72*	**0.73** ^*^	**0.31** ^*^

Particle potency was assessed by lactate dehydrogenase (LDH) release, intracellular ATP, resazurin (RES) reduction, and cytokine (CYT) release after 24 h exposure

*UFP* ultrafine particles, *ALL* regressions performed on data for all size fractions

^*^ *p* < 0.05

^**^ *p* < 0.1

**Fig. 8:**
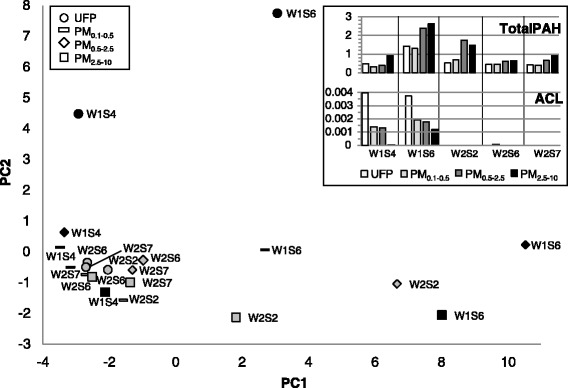
Principal component analysis of size-fractionated particulate matter according to polycyclic aromatic hydrocarbon (PAH) composition. Inset: Mass concentration of total PAHs and acenaphthylene (ACL) per volume of air, ng/m^3^. Black fill, industrial site (W1); grey fill, traffic site (W2)

Assessment of polycyclic aromatic hydrocarbons (PAH) showed that levels tended to be highest in PM_0.5–2.5_ and PM_2.5–10_ fractions (Fig. 8, inset; complete PAH composition and ratios discussed below are provided in Additional file 7: Table S3). The industrial site sample W1S6 exhibited higher PAH levels across size fractions compared to other samples, while sample W2S2 displayed the highest PAH levels of the three samples collected at the traffic site. Industrial site filters contained considerably higher levels of acenaphthylene, which was virtually undetected at the traffic site (Fig. 8, inset), and the ultrafine fraction contained acenaphthene, fluorene, and 2-methylfluorene. Principal component analysis resulted in segregation of the W1S6 industrial site samples from the rest of the group, as well as separation of the ultrafine particles from industrial site sample W1S4, and the traffic site W2S2 PM_0.5–2.5_ and PM_2.5–10_ particles (Fig. 8). Ultrafine samples from the industrial site (W1S4, W1S6) were separated from other ultrafine samples primarily by the second component, driven by naphthalene, acenaphthylene, acenaphthene, fluorene, and methylfluorene. The PM_0.5–2.5_ and PM_2.5–10_ fractions of W1S6 and W2S2 were separated from the group primarily by the first component, with equivalent loadings across a large number of PAHs. The low ratio of B(a)P/B(ghi)P and high ratio of pyrene/B(a)P suggests dominance of automotive combustion sources for the W1S4 samples and W2 samples. The ratio of B(ghi)P/B(e)P, also used as a marker of traffic, was similarly highest for ultrafine and PM_0.1–0.5_ fractions and was higher in ultrafine particles at the traffic site and in industrial site sample W1S4 than in sample W1S6. The ratio of combustion PAHs (Fl, Py, B(a)A, B(b)F, B(k)F, B(a)P, B(e)P, IP) to total PAHs was lowest for industrial site sample W1S4 and highest for industrial site sample W1S6, with samples from the traffic site falling between these two. Total PAHs were not significantly associated with cytotoxicity endpoints (Table 1), but were positively correlated with inflammatory potential (*p* = 0.63, *p* = 0.007), and with the individual cytokines KC (*r* = 0.69, *p* = 0.002), TNF (*r* = 0.62, *p* = 0.008), and IL-6 (*r* = 0.64, *r* = 0.005). However, correlations between PAHs and cytokines did not hold when assessed within each size fraction (Table 1). mRNA levels of the aryl hydrocarbon receptor-regulated gene TiPARP showed a modest association with PAH levels (*r* = 0.49, *p* = 0.046).

**Fig. 9:**
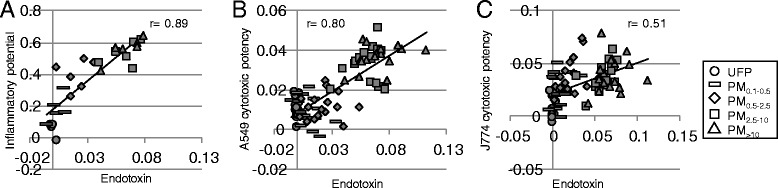
Association of endotoxin with biological effects. Potency estimates were calculated using the results of 3 independent experiments as described in Materials and Methods. Inflammatory potency was calculated as the average of all cytokine potencies, and average cytotoxic potency was calculated as the mean of potencies for lactate dehydrogenase release, resazurin reduction, and ATP content. **a** Inflammatory potency vs. endotoxin. **b** Cytotoxic potency in A549 cells vs. endotoxin. **c** Cytotoxic potency in J774 cells vs. endotoxin. Endotoxin values are presented as EU/mL/μg

Endotoxin levels were significantly higher than field blanks (background) for PM_0.5–2.5_, PM_2.5–10_, and PM_>10_ samples (*Site* × *Size* interaction, *p* = 0.01); however, comparison of data from the three sampling sites showed no evidence of site-specific effects. Endotoxin content increased with particle size (*Size* main effect, *p* < 0.001; UFP < PM_0.1–0.5_ < PM_0.5–2.5_ < PM_2.5–10_ = PM_>10_), and was positively correlated with inflammatory potential (*r* = 0.89, *p* < 0.001; Fig. 9a), driven by effects in the supercoarse and coarse fractions (Additional file 8: Figure S5). Endotoxin levels also correlated with average cytotoxic potency of particles in A549 cells (*r* = 0.80, *p* < 0.001; Fig. 9b), driven by ATP (*r* = 0.73, *p* < 0.001) and LDH (*r* = 0.72, *p* < 0.001), and in J774 cells (*r* = 0.51, *p* < 0.001; Fig. 9c) cells, driven by LDH release (*r* = 0.70, *p* < 0.001). Significant positive correlations were observed for LDH and ATP assays in the PM_2.5–10_ and PM_>10_ fractions (Additional file 8: Figure S5). Resazurin reduction was not consistently correlated with endotoxin levels.

## Discussion

This study compared the relative toxicity of particles collected repeatedly within a small urban area at sites impacted by traffic and industrial sources. Particles from each site and collection period produced dose-related responses for all biological measures in the macrophage-like J774 and epithelial-like A549 cells. The biological potency of particles varied considerably across size fractions, site, and sampling periods, indicating differential toxicity attributable to particle size, composition, and source rather than mass alone. Notwithstanding this variability, systematic differences in the cytotoxic and inflammatory response were evident, including higher potency of coarse particles at the industrial site, and higher potency of ultrafine particles from the traffic site. Regression analyses showed that levels of metals and endotoxin may explain, at least in part, the variation in particle potency observed in these *in vitro* models. Together, the data show that urban particles exhibit considerable temporal variability in potency in relation to differential enrichment of source contributions.

Industrial and traffic emissions are important sources of particles that have been related to human health impacts. An early study by Pope [25] found that hospital respiratory admissions decreased following closure of a steel mill, with aqueous extracts of particles collected during this period producing lower toxicity and inflammatory potential *in vitro* [26] and less pulmonary injury and neutrophilic inflammation *in vivo* [27] compared to particles collected while the steel mill was in operation. A study of children in Windsor found a significant positive association between roadway density around the home and exhaled nitric oxide, a marker of inflammation [28]. A key feature of the present study is the repeated collection of particles at two sites in the vicinity of industrial and traffic sources, in addition to a site representing the urban area background, enabling assessment of site-specific differences and temporal variability. Particles collected to the east of an industrial area that included steel mills and coking operations displayed relatively high levels of metals (including lead) and PAHs during a period in which the prevailing wind was from the west (W1S6), as compared to a southerly flow (W1S4) that was more likely associated with regional background air. Certain PAHs detected on filters at the industrial site, notably acenaphthylene, acenaphthene, fluorene, and 2-methylfluorene, have a large fraction in the gas phase, and so levels may be artifacts of the collection process rather than reflecting particle composition. Particles collected at the traffic site displayed elevated levels of zinc and barium, trace metals associated with vehicle emissions (tire and brake lining wear, lubricating oil additives) during the W2S6 sampling period that produced the highest inflammatory potency of the fine PM_0.1–0.5_ and PM_0.5-2.5_ fractions. However, vanadium, an indicator of fossil fuel refining and/or oil burning processes, was also increased during the W2S6 period, indicating contributions of other emission sources. Overall, the site-dependent contrasts and considerable variability in potency and composition is consistent with contributions from multiple sources, which is to be expected with time-integrated samples.

We took a two-step approach to compare the biological responses to particles according to the premise that effects attributable to sources or particle constituents may or may not be modified by particle size. We first analysed data from all size-fractions together to explore possible relationships between site of collection and potency and any interactions with particle size. On the basis of significant *Site* × *Size* factor interactions, we then examined the relationship of particle composition with potency within each size-fraction. Comparison of particle toxicity on an equivalent mass concentration basis revealed site-dependent effects that were size-specific: larger particles (PM_2.5–10_, PM_>10_) were more potent at the industrial site (according to LDH release, ATP levels, and inflammatory response), while ultrafine particles were more potent at the traffic site (according to resazurin reduction). PM_>10_ samples possibly included resuspended dust, and may have been less sensitive to the day-to-day fluctuations in composition and potency that could occur with smaller size-fractions as a result of changing environmental conditions and source contributions. These particles might therefore serve as a more stable indicator of site-specific differences over time. Impacts of changes in wind direction on source enrichment were expected to be more pronounced at the industrial site compared to the traffic site, as sampling was conducted at a distance from the industrial sources, and this was reflected in the contrasting metal composition of industrial site samples collected over distinct periods. On the other hand, the higher cytotoxic potential of ultrafine particles from the traffic site, associated with elevated levels of metal and PAH tracers of traffic emissions, suggests that enrichment of fresh traffic emissions may be driving this effect. Some have observed a greater inflammatory response to fine traffic-derived particles compared to particles from other urban sources [11, 29], while others have not [12]. The considerable variability in cytotoxic and inflammatory potential of particles collected at the “traffic” site, which likely include contributions by other sources, illustrates the complexity of source attribution in environmental sampling. Importantly, endotoxin levels did not differ among sites, nor was there any association between endotoxin levels and inflammatory responses in the ultrafine or fine fractions. It is therefore unlikely that endotoxin was responsible for site differences in biological response. Inflammatory cytokines did, however, correlate with endotoxin in coarse particles, as has been seen in other studies [11, 12, 30].

The importance of metals in mediating cytotoxicity and inflammatory effects of particles has been demonstrated through *in vitro* and *in vivo* studies [31–33]. In a recent panel study conducted near a steel mill, metal constituents of PM_2.5_ were associated with increased heart rate and blood pressure and decreases in measures of lung function [34]. Such findings substantiate population studies relating hospital admissions and mortality to specific metal constituents [35, 36]. Data from the present study showing particle size-dependent associations of metals with metabolic function (as assessed by resazurin reduction) and with transcript levels of the metal-responsive gene metallothionein-2, suggest that chemical and physical properties of the particles are involved in generating these responses. As nanosized particles are poorly sensed and less efficiently internalised by cells [37], size-dependent differences in response may relate to the cellular capacity to sense the material, or to its bioaccessibility under these cell culture conditions. Although soluble metal content has been related to the toxicity of ambient particles, dissolved metal ions in particle-free exposures can produce markedly lower cytotoxicity *in vitro* than is seen with whole particle exposures, and particles can increase exposure to metals by facilitating internalisation and intracellular solubilisation [38]. The relative contribution of soluble and insoluble metals to these effects warrants further study, as size-dependent effects may relate to differential solubility of metals across size fractions.

The primary goal of the present work was to use separate samples collected on multiple days in the vicinity of specific emission sources to test for systematic differences in biological potency related to enrichment of source contributions. Strengths of this study include the repeated sampling approach that allowed assessment of temporal and spatial variability, comparison of the potency of five particulate size fractions, and the use of two cell lines and multiple assays to generate a rich dataset for analysis of effects and regression against constituents. The variability in potency across sites, sampling periods, and particle size revealed by the repeated sampling provided a basis for assessing the relationship between potency and specific physicochemical characteristics, notably particle size, metals, and endotoxin. One of the challenges of collecting size-fractionated particles over relatively short sampling periods is the small amount of material collected, which limits the number and type of analyses that can be performed. Chemical analyses were geared towards characterisation of potential source contributions, and so analyses were performed on filter segments using accepted approaches (i.e. solvent and acid-based extractions) for characterisation of environmental atmospheres rather than on the particulate matter sequentially extracted by sonication in methanol and water that was used for toxicity testing. This approach enabled use of the small amount of material available to assess contrasts in the cytotoxic and inflammatory potential of collected particles impacted by different sources, as well as to examine metal and PAH speciation that is consistent with standard atmospheric sampling. A limitation of this approach is the resulting uncertainty in relating biological effects to the composition of the exact material to which the cells were exposed. Metabolic activity was closely associated to metal content, particularly in PM_0.5–2.5_ and PM_2.5–10_ fractions, suggesting that the relative proportions of metals may have been maintained through extraction processes. The general lack of association between PAH levels and toxicity endpoints should not be taken as evidence that PAHs have no effect, as volatility of organic species during filter processing likely resulted in alteration of the overall profile. Similarly, the lower extraction efficiency of UFP from polypropylene filters compared to larger particles from PUF substrates could contribute to the lack of association of metals with biological endpoints for this size fraction. In a future study conducted using particulate samples collected over a longer period and with considerably higher extracted mass we will examine metal and PAH content of the same particulate matter used in the *in vitro* assays.

Biological effects tend to correlate well with particle number and surface area when comparing particles of similar chemical composition, emphasising the importance of considering fine and ultrafine particles in health impacts; however, as found in this study, *in vitro* studies of ambient particles often find the coarse fraction to be the most toxic or inflammogenic [12, 30, 39]. We employed macrophage and epithelial cell lines and a panel of toxicological assays to assess responses to particle exposure with the intention of capturing possible complexity in the response and to yield a more detailed characterisation of particle toxicity than would be revealed by a single assay or cell type. The screening platform was clearly sensitive to several important parameters of particle toxicity, notably ambient particle size, metal content, and endotoxin (Table 1).

In general, the macrophage-like J774 cell line was more responsive to particles, to site differences, and to metals compared to the epithelial A549 cells, in line with previous work [40]. It is noteworthy that results from three assays used to assess cell viability differed, suggesting sensitivity to distinct particle constituents or characteristics. While potency according to cell membrane integrity (as measured by LDH release), energy metabolism (intracellular ATP), and inflammatory cytokine secretion were positively correlated with particle size and endotoxin content in both cell types, metabolic activity measured by resazurin reduction exhibited a distinct pattern of response by cell type and size that correlated well with metal content. The compound resazurin is used extensively as an indicator of cellular metabolic status and health, and is reduced in living cells by mitochondrial and cytoplasmic reductases to the fluorescent product resorufin [41]. The remarkably strong correlation between metals and decreased production of resorufin suggests that cellular reductive capacity was a more selective indicator of metal content than LDH release or ATP, and was not strongly influenced by endotoxin. Cytotoxicity is a distal endpoint that reflects the combined actions of physical stressors associated with cell size, surface area, or particle number, as well as the effects of bioaccessible particle fractions on a number of biological pathways and processes. Clearly, it is important to assess multiple indices of toxicity, as particle potency will depend on the model and assays employed in addition to the inherent properties of the particles. Moreover, as cytotoxicity may not necessarily correlate with other biological responses (e.g. respiratory burst [42]), responses in a given set of assays may only be relevant to a subset of health outcomes.

## Conclusions

The complex nature of ambient PM presents challenges in elucidating drivers of toxicity beyond mass and aerodynamic diameter. Refinement of current air quality guidelines to target those emissions most relevant to human health effects will require a greater understanding of the relative importance of physicochemical characteristics and constituents in eliciting biological responses relevant to adverse health outcomes in humans. We found that particles collected repeatedly in the vicinity of traffic and industrial sources exhibited potency contrasts according to size, sampling site, and period of collection in an *in vitro* model. The relatively large number of samples collected within a small urban area enabled assessment of variability in potency across time, with composition and potency changing in relation to wind direction and source contributions. Together, the data show that 1) day-to-day fluctuations in source contributions can significantly impact particle composition and potency in biological systems; 2) industrial and traffic emission sources contribute differentially to the cytotoxic and inflammatory potency of specific particulate size-fractions; and 3) particle constituents may elicit distinct biological responses, with cellular metabolic activity closely associated with total metal content. Importantly, we found that conventional cytotoxicity assays are sensitive to distinct particle characteristics and constituents, suggesting that use of multiple assays should be considered in characterising impacts of particles on cell viability. Investigation of the potency of source-specific particulate matter using *in vitro* and *in vivo* models should contribute to meaningfully interpreting epidemiological observations of effects in the human population (e.g. increased incidence of asthma in proximity to roadways), and in assisting efforts to prioritise sources and particle constituents for regulatory action.

## Materials and methods

### Particle collection

Size-fractionated particles were collected at three sites (industrial, traffic, urban background; Fig. 1) within Windsor, Ontario using a ChemVol High Volume Cascade Impactor [43]. This sampler consists of several impaction stages with PUF collection surfaces followed by collection of ultrafine particles on polypropylene filters. Five stages were utilized, providing samples of particles >10 μm in aerodynamic diameter (PM_>10_), between 2.5 and 10 μm (PM_2.5–10_), between 0.5 and 2.5 μm (PM_0.5–2.5_), between 0.1 and 0.5 μm (PM_0.1–0.5_) and <0.1 μm (ultrafine particles). A high capacity Roots™ Universal RAI Rotary Positive Blower was used to maintain the highest flow rate possible through the ChemVol impaction slits. The main restriction and greatest pressure drop was provided by the 0.1 μm cut stage, which limited the flow rate to the design flow of about 900 L/min according to the manufacturer (Rupprecht and Patashnick, Albany, NY). The sample collection period spanned Aug. 2–Oct. 14, 2005 and Mar. 29-Oct. 6, 2006. Particles were collected over 7 sampling periods of 2–4 days at both the industrial (W1S1-W1S7) and traffic (W2S1-W2S7) sites, and two periods at the urban site (W3S1, W3S2). The W3 sampling periods were interrupted by vandalism at the site, resulting in uncertainty in the duration of the collection period for W3S1. Field PUF/filter blanks were also included in the analyses (W4S1 and W4S2). Wind roses were generated for each sampling period using meteorological data collected by Environment Canada (http://weather.gc.ca) at Windsor Airport.

### Particle extraction and resuspension for toxicity studies

Filters were sectioned for extraction of particles for toxicity studies and chemical analyses. Particles were sequentially extracted by sonication for 60 min in methanol and twice more in nanopure water. The eluted material was combined and concentrated by lyophilization, resuspended in water, aliquoted in vials, and dried by lyophilization. Extraction from particle collection substrates can release PUF or filter material that contributes to the extracted mass. UFP extracts were passed sequentially through a sterile 40 μm nylon filter (BD Falcon Cell Strainer, Corning Life Sciences, Corning, NY) and a 5 μm filter (Target Syringe Filter, National Scientific Co., Rockwood, TN) to remove fibres derived from the polypropylene filters. Extraction efficiencies from PUFs and polypropylene filters using this approach are on the order of 90 and 40 % respectively. Debris from the extraction process assessed by extraction of blank (unexposed) PUFs and polypropylene filters contribute 5–10 % of extracted mass, and so should not significantly impact comparisons of particles at equivalent mass concentrations. Analysis of blank PUF and polypropylene filter extracts for metals showed that the concentrations of all elements were below minimal detectable levels. Blank filter and PUF extracts were compared alongside particles for all toxicity experiments to test for any biological effects of PUF and filter residues. Standard reference materials (SRM) TiO_2_ (SRM-154b) and SiO_2_ (SRM-1879a) were obtained from the National Institute of Standards and Technology (NIST, Gaithersburg, MD). Preparation and characterisation of the urban particulate matter standard EHC-6802 was described previously [44]. Particles were resuspended at 10 mg/mL in saline (1.9 mg NaCl/mL) supplemented with 25 μg/mL of the non-ionic detergent Tween 80. The suspensions were vortexed for 30 s, sonicated for 20 min in an ice-cold water bath and dispersed by 25 strokes of the homogenizer piston in a Dounce glass-glass micro-homogenizer. Particle suspensions were aliquoted into sterile microcentrifuge tubes and heated to 56 °C for 30 min [45]. Stock suspensions were stored at −80 °C until use.

### Chemical characterisation of particles

#### Elemental analysis

PUF substrates and ultrafine polypropylene filters were sectioned at a Class 100 HEPA clean bench (Labconco Corporation, Missouri, USA) using stainless steel blades. Multi-element spikes and the SRM-1649 (NIST, Gaithersburg, MD, USA) were added to blank PUF substrates and filters and processed together with samples. All samples were digested in a closed vessel microwave assisted reaction system (MARS 5, CEM Corporation, Matthews, NC) using 100 mL Teflon liners (XP-1500, CEM Corporation, Matthews, NC). Samples and substrates were dissolved as previously described [46]. After digestion, all samples were evaporated to near dryness in the presence of concentrated HCl [47] and diluted to 15 mL with 2 % (*v/v*) HCl prior to ICP–MS analysis. All measurements were performed using a 7500ce ICP–MS system (Agilent Technologies, Wilmington, DE, USA). The octopole collision/reaction system (ORS), was pressurized with He gas for analysis of V, As and Cr, and with H_2_ for analysis of Fe and Se. Internal standardization with 0.5 mg/L solution of ^45^Sc, ^89^Y, ^115^In, and ^165^Ho was used to correct for the instrumental drifts and nonspectral interferences. Quality control samples were used to determine the accuracy and precision of chemical analysis, and to diagnose contamination. Air concentrations of each element were determined by dividing the blank corrected element mass per PUF/filter section by the air volume sampled through the ChemVol.

#### Organic analysis

PUF ring and filter sections were spiked with 50 μL of isotopically labeled PAH surrogate standards for recovery correction. Samples were extracted by Soxhlet apparatus in 350 mL of cyclohexane for 16 to 20 h. Following evaporation to ~5 mL, the extract was subjected to activated silica gel column chromatography for fractionation of the desired target analytes using a suite of solvents of increasing polarity. First, the column was pre-washed with 10 mL of hexane, then eluted with 5 mL of hexane followed by 5 mL of benzene, and the fraction was archived (aliphatic hydrocarbon fraction). The PAHs were then eluted in the second fraction with two 5 mL aliquots of benzene into a calibrated centrifuge tube. The benzene fraction was concentrated to ~0.5 mL by ultra-high purity nitrogen. After addition of 50 μL d10 fluoranthene (10 ng/μL), the purified sample extract was reconstituted to 0.5 mL using benzene prior to GC/MS analysis. The purified extract was analysed for PAHs using low resolution Agilent 7890A GC interfaced directly to Agilent 5975C Mass Selective Detector under the following operating conditions: 1 μL sample injection using Agilent 7693 autosampler in splitless mode at 280 °C; chromatographic column: 30 m DB XLB fused silica, 0.25 mm ID and 0.25 μm film thickness using helium carrier gas with constant flow of 2.0 mL/min. GC oven temperature was programed as follows: an initial temperature of 90 °C for 4 min, ramp to 200 °C at 20 °C/min, 250 °C at 2.5 °C/min, then to 283 °C at 1.5 °C/min and hold for 6 min. The detection mode used was electron impact ionization @ 70 eV operated in the Selected Ion Monitoring (SIM) mode.

#### Endotoxin

Endotoxin levels were assessed using the Limulus Amebocyte Lysate (LAL) chromogenic quantitation kit (Lonza, Walkersville, MD, USA) with a Synergy 2 multi-mode plate reader (Bio-Tek, Winooski, VT, USA) according to manufacturer instructions, with the following modification: following addition of the stop reagent, samples were centrifuged to pellet insoluble material that could interfere with the assay. The clarified supernatant was transferred to a new plate for measurement of absorbance.

#### *In vitro* exposures

Human lung epithelial (A549; ATCC, CCL-185) and murine macrophage (J774A.1; ATCC, TIB-67) cell lines (American Type Culture Collection, Manassas, VA, USA) were propagated in Dulbecco’s Modified Eagle’s Medium (Fisher Scientific) containing phenol red (excluded for particle exposures and bioassays) and 4.5 g/L glucose and supplemented with FBS (10 % *v/v*, non-heat inactivated; Fisher Scientific) and gentamicin (50 μg/ml; Sigma-Aldrich Canada, Oakville, ON) in 75 cm^2^ tissue culture flasks (Corning, NY, USA) at 37 °C, 5 % CO_2_, and 95 % relative humidity. Cells were seeded in 96-well black-walled clear-bottom cell culture plates (BD Biosciences, Mississauga, ON) at 100 μL/well (A549, 1 × 10^4^ cells/well, 3 × 10^4^ cells/cm^2^; J774A.1, 2 × 10^4^ cells/well, 6 × 10^4^ cells/cm^2^) of complete medium, and incubated at 37 °C for 24 h. Particle suspensions were thawed, sonicated for 20 min in an ice-cold ultrasonic water bath, diluted in complete media devoid of serum, and sonicated for a further 5 min immediately prior to cell dosing. Cell monolayers were exposed to 100 μl particle suspensions resulting in 0, 30, 100, and 300 μg/cm^2^ in a final volume of 200 μl. Doses were selected to range from levels that do not cause measurable cytotoxic responses to ones that do result in cytotoxicity so that dose-response relationships could be evaluated. In order to enable assessment of early events that precede pronounced cytotoxicity as well as later cytotoxic effects, cells were incubated at 37 °C for 4 h (mRNA and cytokine assays in J774A.1 cells) and 24 h (cytotoxicity assays in A549 and J774A.1 cells) respectively. For gene expression analyses the doses were extended to include 0.01, 0.1, 1, and 10 μg/cm^2^. Three independent experiments were conducted for each cell line.

#### Assessment of cytotoxic and inflammatory responses

Supernatant (100 μL) was transferred into a clear, conical bottom 96-well plate (VWR, Mississauga, ON) and clarified by centrifugation at 350 × g for 5 min for use in the LDH and inflammatory cytokine assays. Metabolic activity was assessed using aliquots collected from the cell supernatant immediately and 2 h after addition of the resazurin reagent mixture. Aliquots were transferred into clean plates containing 80 μL of serum-free complete medium per well, shaken at 350 rpm for 30 s on a circular plate shaker, and clarified by centrifugation at 300 × g. Reduction of the dye resazurin to resorufin was calculated by fluorescence at 2 h minus baseline fluorescence measured by top-reading at λEx = 540 nm and λEm = 600 nm (Synergy 2). After removal of the supernatant, cells were lysed in 100 mM MgCl_2_ and 0.025 % Triton X-100 in PBS at room temperature for 10 min. The lysate was transferred into clean 96-well conical bottom plates and clarified by centrifugation for 5 min at 350 × g for use in LDH and ATP assays. LDH levels were measured using the CytoTox 96 colorimetric assay (Promega Corporation, Madison, WI) at an absorbance of 490 nm (Synergy 2) following incubation for 20 and 40 min (supernatant) and 0 and 10 min (lysate), and calculated as a fraction of total LDH activity recovered in supernatant and cell lysate. Cellular ATP content was assessed using the ViaLight Plus assay (Lonza Corporation, Rockland, ME) using the Synergy 2. For mRNA analyses cells were lysed with TRIzol (Life Technologies) and RNA was extracted with Direct-zol-96 RNA kits (Cedarlane Laboratories, Burlington, Ontario, Canada). Levels of IL-1α, IL-6, IL-10, IL-13, G-CSF, RANTES, TNF, and KC were assessed in J774 cell supernatants using an 8-plex murine cytokine assay panel (Bio-Rad Laboratories (Canada) Ltd., Mississauga, Ontario, Canada) on a Bio-Plex 200 multiplex luminescence assay system (Bio-Rad Laboratories (Canada) Ltd.).

#### Gene expression analyses

Primers were designed to produce amplicons of less than 150 bases and an optimal annealing temperature of 60 °C using the Universal Probe Library design software (Roche Diagnostics Canada, Laval, Quebec, Canada) and Primer-BLAST software (National Center for Biotechnology Information, Bethesda, MD), and validated for high (>90 %) efficiency (Additional file 9: Table S4). First strand synthesis was performed using the High-Capacity cDNA Reverse Transcription Kit with RNase Inhibitor (Life Technologies Inc., Burlington, Ontario, Canada). Real-time PCR was conducted using iQ SYBR Green Supermix (Bio-Rad Laboratories (Canada) Ltd, Mississauga, Ontario, Canada) and 200 nM primer in 384-well plates using a spectrofluorometric thermal cycler (Lightcycler 480, Roche Diagnostics Canada). Negative control samples from the reverse transcription reaction were evaluated to test for the presence of contaminating genomic DNA, and a melt curve was conducted following each run to verify product purity. Expression was calculated relative to β-actin using the delta-delta Ct method [48], and expressed as fold change relative to the mean of all control (0 μg/cm^2^) samples.

#### Potency estimates

Cytotoxicity endpoints (lactate dehydrogenase release, resazurin reduction, ATP content), cytokine release, and mRNA data were normalized to the mean of the respective controls to generate fold-change values for each particle dose. As a simplified description of the dose-effect relationship, potency estimates (β) were derived from the following equation: fold-change = (Dose + 1)^β^ where β is the slope of the dose-effect relationship on the logarithmic scale [24]. Dose-effect data were fitted using CurveExpert v1.3 (D. Hyams, Hixson, TN, USA). To enable averaging, resazurin reduction and ATP content potencies (where a negative β indicates greater potency) were multiplied by −1 so that for all assays higher β indicated higher potency.

#### Statistical analyses

Cytotoxicity and cytokine dose-response data were assessed for statistically significant effects by three-way ANOVA with *Dose* (0, 30, 100, 300 μg/cm^2^), *Site* (W1, W2, W3) and *Size* (PM_<0.1_, PM_0.1–0.5_, PM_0.5–2.5_, PM_2.5–10_, PM_>10_) as factors. Potency data were assessed by two-way ANOVA with *Site* and *Size* as factors. Datasets not meeting the assumptions of normality and equal variance for ANOVA were rank transformed prior to analyses. Pairwise multiple comparisons were carried out using the Holm-Sidak procedure to elucidate the pattern of significant effects (α = 0.05). Correlations were performed between toxicological endpoints and particle chemistry using Pearson’s correlation coefficient. All statistical analyses were conducted using SigmaPlot version 12 (Systat Software, Inc., San Jose, CA, USA). Clustering of particles and principal component analyses based on their elemental and PAH composition were conducted using Minitab, version 15 (Minitab Inc., State College, PA, USA). Heatmap software was used to visualise data (http://www.hiv.lanl.gov/content/sequence/HEATMAP/heatmap_mainpage.html; Los Alamos National Laboratory, Los Alamos, NM, USA).

## Additional files

Additional file 1: Figure S1. Wind roses for all sampling periods. Wind roses are labelled according to sampling period (e.g. W1S1 indicates sample 1 collected at site W1). Vectors represent the proportion of a collection period that the wind was coming from a given direction. W1, industrial site; W2, traffic site; W3, urban background site. Additional file 2: Figure S2. Cytotoxic responses to 24 h particulate matter exposures in J774A.1 cells. Cell exposures and assays were conducted as described in the Materials and Methods (*n* = 3 independent experimental repeats). A) ATP content. B) Metabolic reduction of non-fluorescent resazurin. C) Lactate dehydrogenase (LDH) release. Values are presented as average fold-change over control ± standard error (n = 3 experimental repeats). Additional file 3: Figure S3. Cytotoxic responses to 24 h particulate matter exposures in A549 cells. Cell exposures and assays were conducted as described in the Materials and Methods (n = 3 independent experimental repeats). A) ATP content. B) Metabolic reduction of non-fluorescent resazurin. C) Lactate dehydrogenase (LDH) release. Values are presented as average fold-change over control ± standard error (n = 3 experimental repeats). Additional file 4: Table S1. Table of significant effects identified through statistical analysis of the cytotoxicity data. Cytotoxicity data was analysed by three-way ANOVA (factors *Dose*, *Site*, and *Size*), and potency data was analysed by two-way ANOVA (factors *Site* and *Size*), followed by Holm-Sidak pairwise comparison where appropriate, as described in the Materials and Methods. Additional file 5: Figure S4. Hierarchical clustering of particles according to mRNA response in J774 cells. Potencies were calculated using the results of 3 independent experiments. The heat map displays particle potency for all transcripts assayed that were within the limits of detection. Descriptions of the main contents of each cluster are included to the left of the plot. Red indicates increased expression, green indicates decreased expression. W1, industrial site; W2, traffic site; W3, urban background site; W4, field blanks; TiPARP, TCDD-inducible poly (ADP-ribose) polymerase; MT, metallothionein; ET, endothelin; HMOX, heme oxygenase; TNF, tumour necrosis factor; iNOS, inducible nitric oxide synthase; IL6, interleukin-6. Additional file 6: Table S2. Table of metal content of samples collected in Windsor, Ontario. Metals were analysed as described in the Materials and Methods. Data are sorted according to site and size fraction, and presented as airborne concentration (ng/m^3^). Additional file 7: Table S3. Table of polycyclic aromatic hydrocarbon (PAH) content of samples collected in Windsor, Ontario. PAHs were analysed as described in the Methods section. Data are sorted according to site and size fraction, and presented as airborne concentration (ng/m^3^). Additional file 8: Figure S5. Association of cytotoxic potency with endotoxin content in size-fractionated particles after 24 h exposure of J774A.1 and A549 cells. A) Lactate dehydrogenase (LDH) release vs. endotoxin. B) Resazurin reduction vs. endotoxin. C) ATP content vs. endotoxin. D) Cytokine release vs. endotoxin. Endotoxin values are presented as EU/mL/μg. Additional file 9: Table S4. Table of primers used for gene expression analyses. Primers were designed for real-time polymerase chain reaction assays as described in the Materials and Methods.
