# Supplementary figures and images for: Cytotoxic and inflammatory potential of size-fractionated particulate matter collected repeatedly within a small urban area
Source: Part Fibre Toxicol. 2015 Jul 16;12:24. doi: 10.1186/s12989-015-0099-z (PMC4502610; doi:10.1186/s12989-015-0099-z)

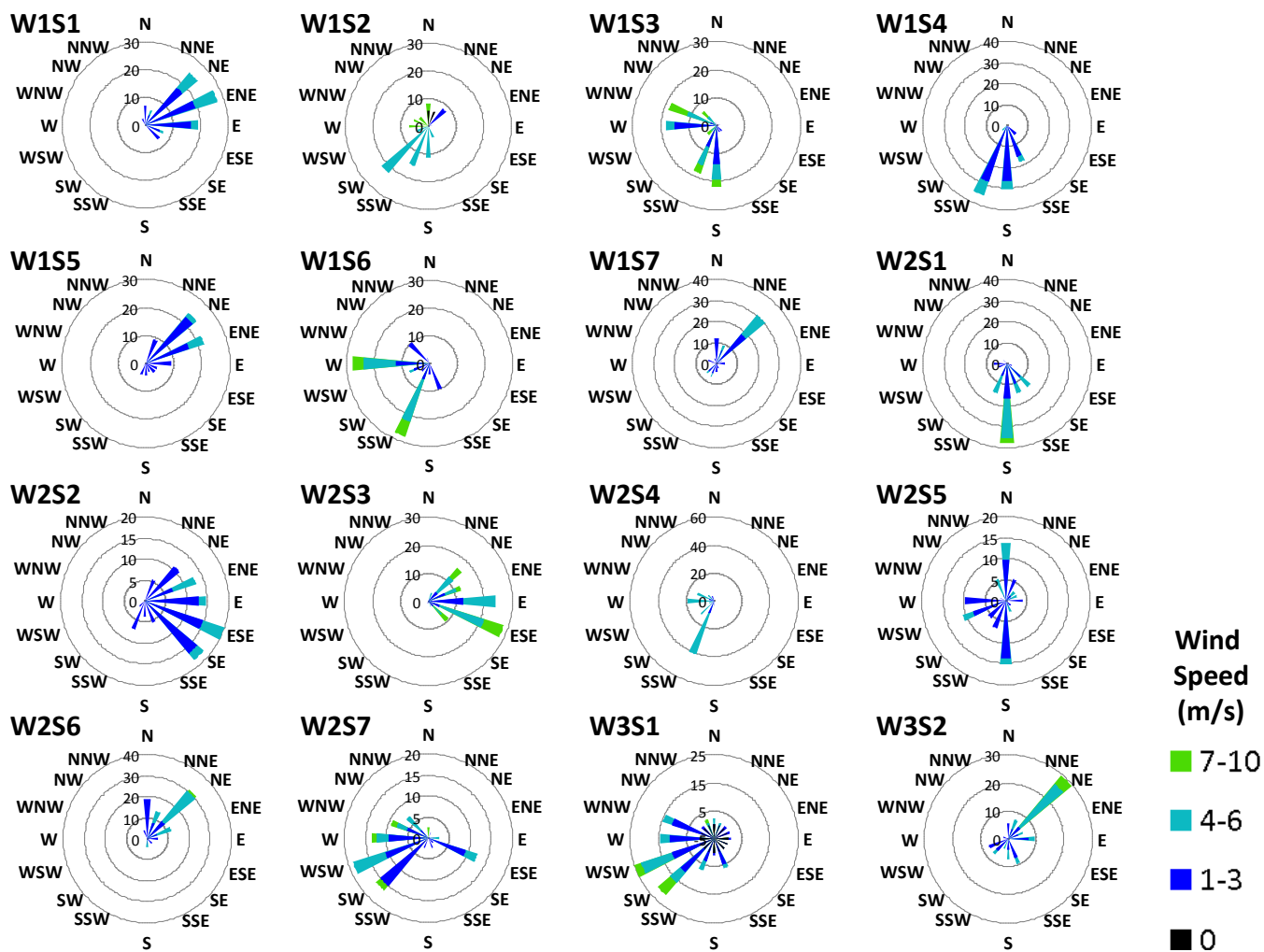

Supplement: Additional file 1: Figure S1. — Wind roses for all sampling periods. Wind roses are labelled according to sampling period (e.g. W1S1 indicates sample 1 collected at site W1). Vectors represent the proportion of a collection period that the wind was coming from a given direction. W1, industrial site; W2, traffic site; W3, urban background site. [file 12989_2015_99_MOESM1_ESM.pdf]

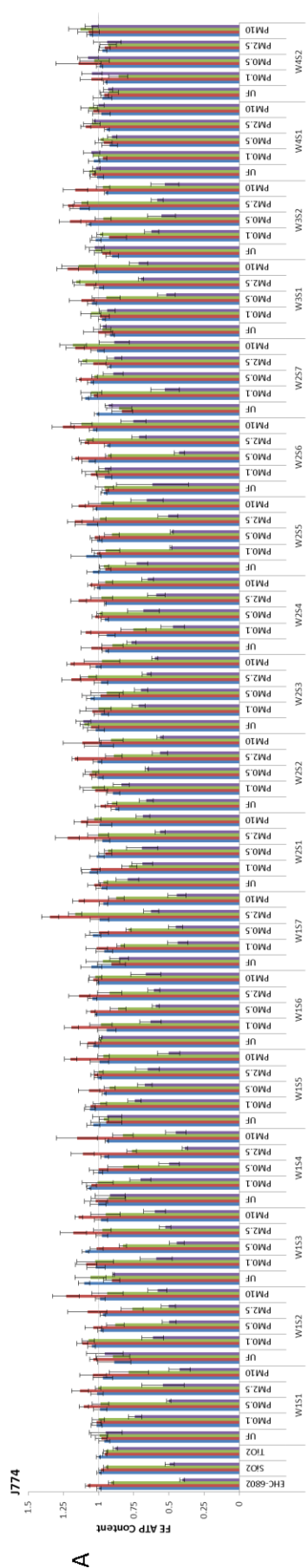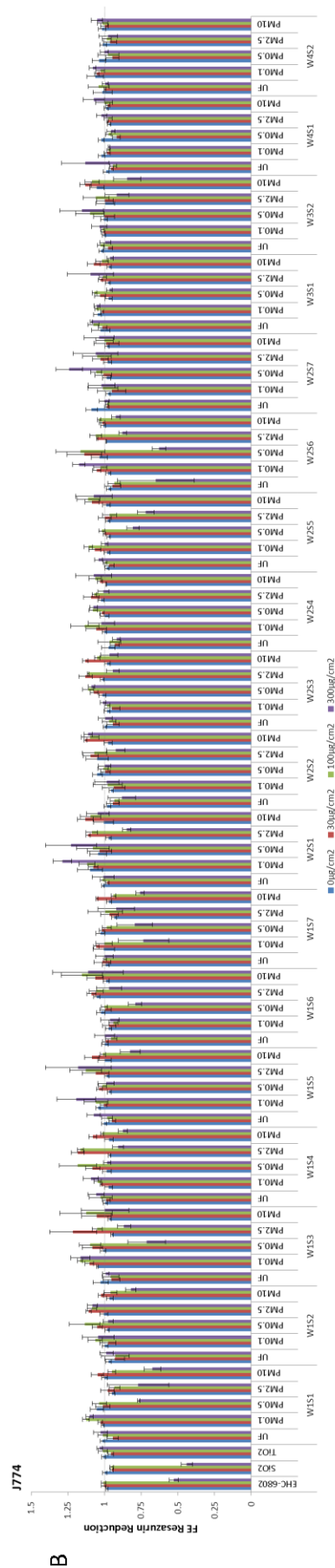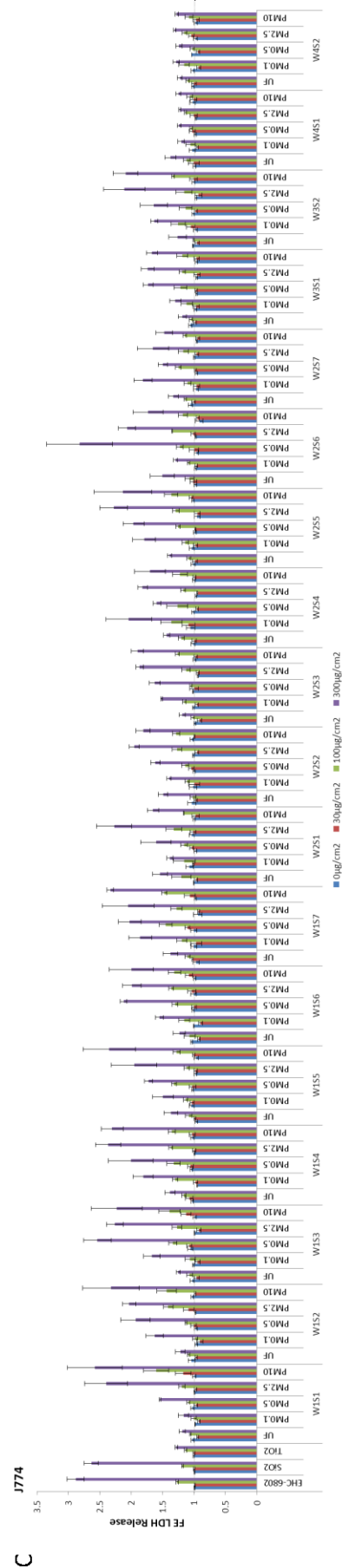

Supplement: Additional file 2: Figure S2. — Cytotoxic responses to 24 h particulate matter exposures in J774A.1 cells. Cell exposures and assays were conducted as described in the Materials and Methods (n = 3 independent experimental repeats). A) ATP content. B) Metabolic reduction of non-fluorescent resazurin. C) Lactate dehydrogenase (LDH) release. Values are presented as average fold-change over control ± standard error (n = 3 experimental repeats). [file 12989_2015_99_MOESM2_ESM.pdf]

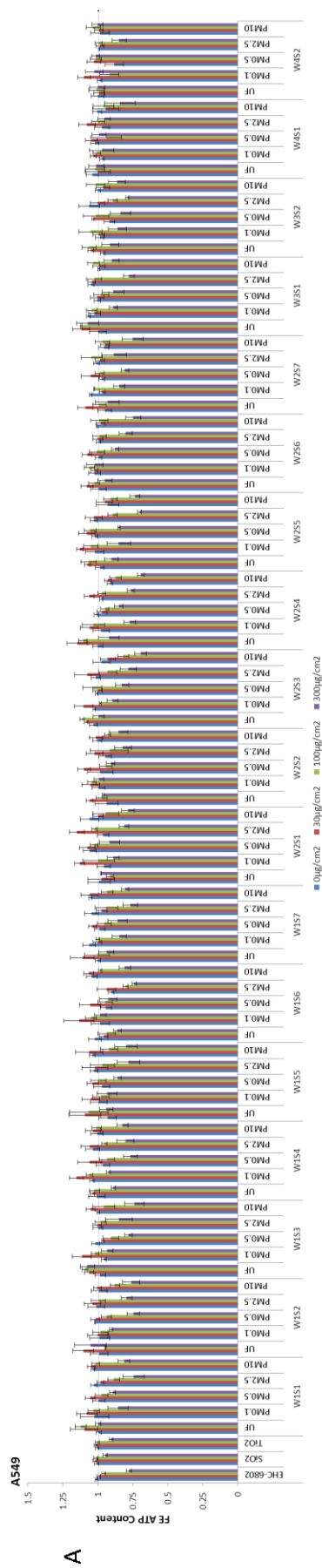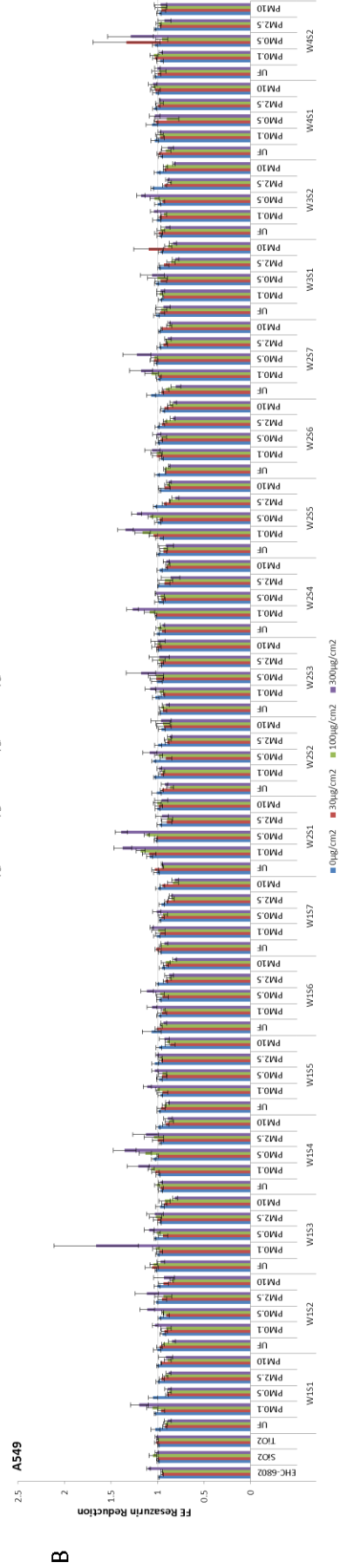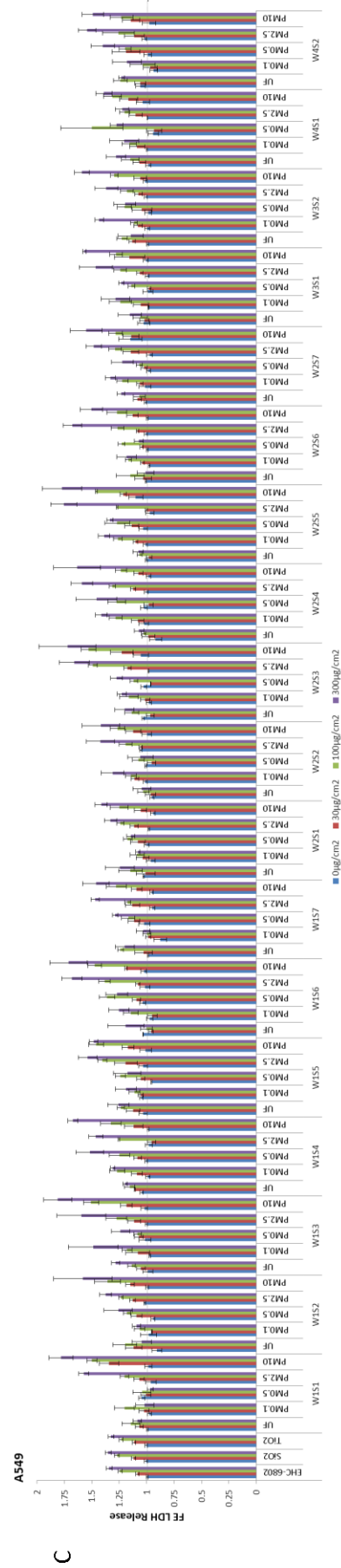

Supplement: Additional file 3: Figure S3. — Cytotoxic responses to 24 h particulate matter exposures in A549 cells. Cell exposures and assays were conducted as described in the Materials and Methods (n = 3 independent experimental repeats). A) ATP content. B) Metabolic reduction of non-fluorescent resazurin. C) Lactate dehydrogenase (LDH) release. Values are presented as average fold-change over control ± standard error (n = 3 experimental repeats). [file 12989_2015_99_MOESM3_ESM.pdf]

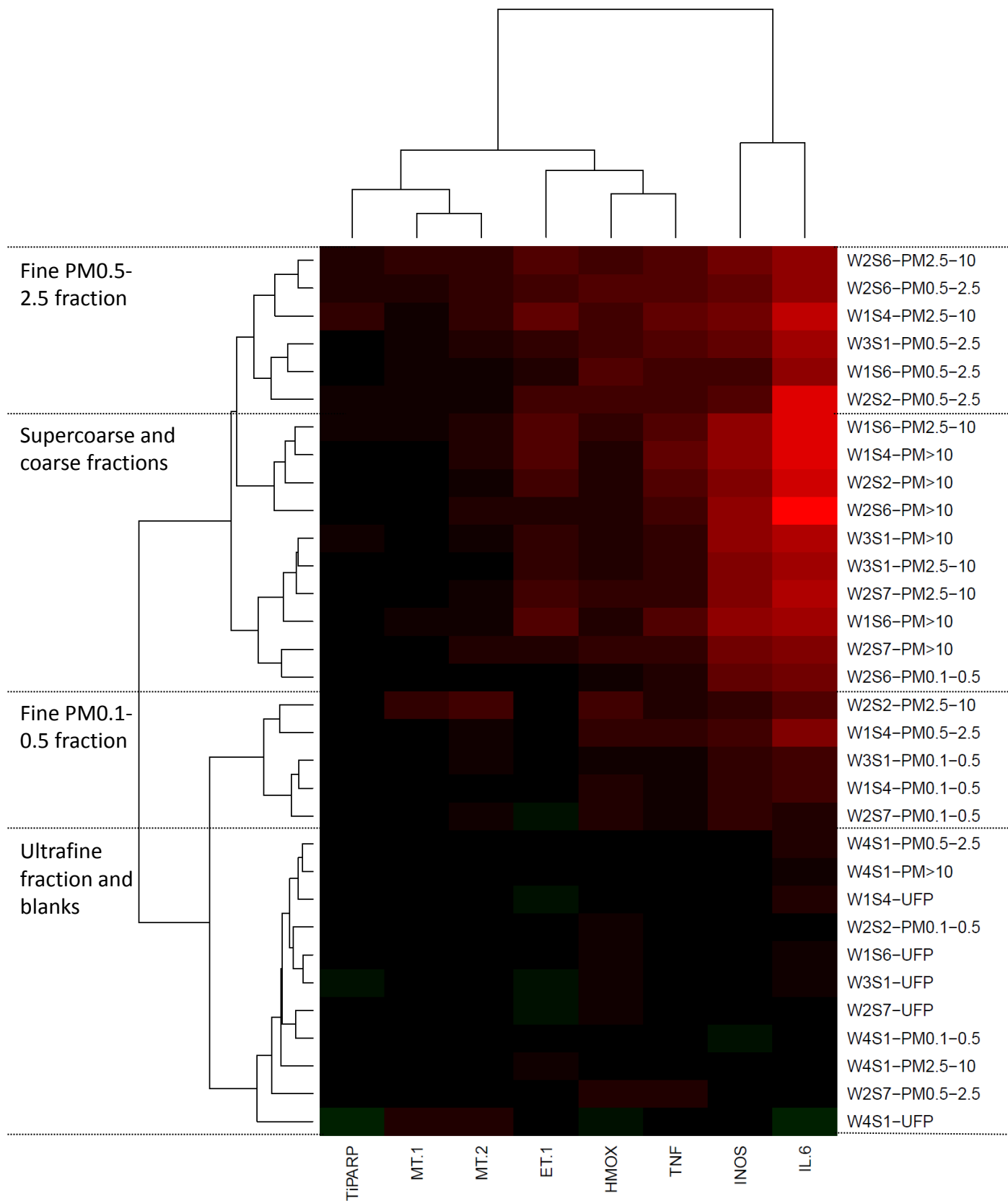

Supplement: Additional file 5: Figure S4. — Hierarchical clustering of particles according to mRNA response in J774 cells. Potencies were calculated using the results of 3 independent experiments. The heat map displays particle potency for all transcripts assayed that were within the limits of detection. Descriptions of the main contents of each cluster are included to the left of the plot. Red indicates increased expression, green indicates decreased expression. W1, industrial site; W2, traffic site; W3, urban background site; W4, field blanks; TiPARP, TCDD-inducible poly (ADP-ribose) polymerase; MT, metallothionein; ET, endothelin; HMOX, heme oxygenase; TNF, tumour necrosis factor; iNOS, inducible nitric oxide synthase; IL6, interleukin-6. [file 12989_2015_99_MOESM5_ESM.pdf]

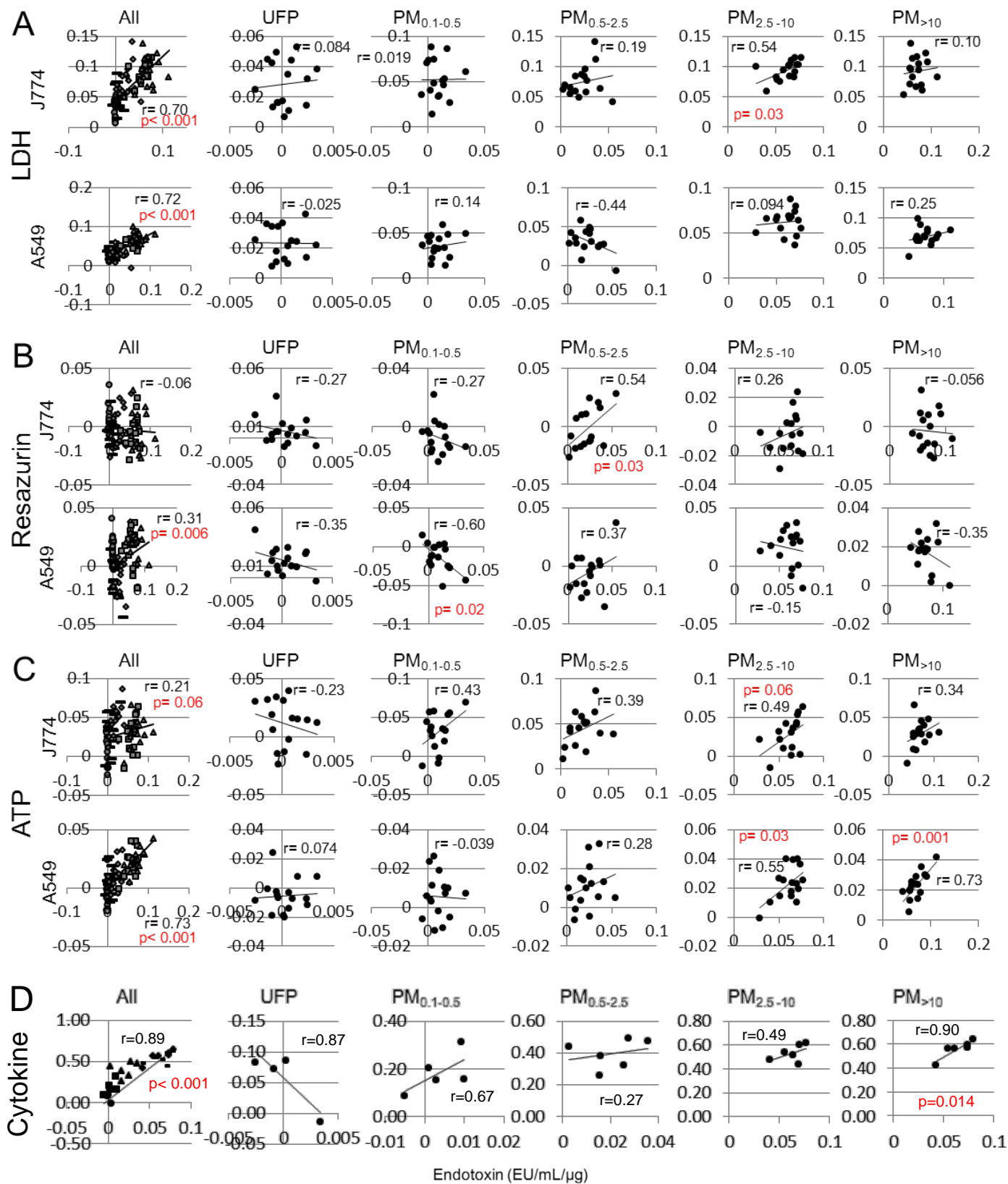

Supplement: Additional file 8: Figure S5. — Association of cytotoxic potency with endotoxin content in size-fractionated particles after 24 h exposure of J774A.1 and A549 cells. A) Lactate dehydrogenase (LDH) release vs. endotoxin. B) Resazurin reduction vs. endotoxin. C) ATP content vs. endotoxin. D) Cytokine release vs. endotoxin. Endotoxin values are presented as EU/mL/μg. [file 12989_2015_99_MOESM8_ESM.pdf]
